# Impaired Nutritional Condition After Stroke From the Hyperacute to the Chronic Phase: A Systematic Review and Meta-Analysis

**DOI:** 10.3389/fneur.2021.780080

**Published:** 2022-02-01

**Authors:** Viviënne Huppertz, Sonia Guida, Anne Holdoway, Stefan Strilciuc, Laura Baijens, Jos M. G. A. Schols, Ardy van Helvoort, Mirian Lansink, Dafin F. Muresanu

**Affiliations:** ^1^Department of Respiratory Medicine, Maastricht University, Maastricht, Netherlands; ^2^Danone Nutricia Research, Utrecht, Netherlands; ^3^DHealth, Consultant Dietitian, BMI/Circle Bath Clinic, Education Officer for the British Association for Parenteral and Enteral Nutrition and Chair of the UK Managing Adult Malnutrition in the Community Panel, Bath, United Kingdom; ^4^Department of Neurosciences, “Iuliu Hatieganu” University of Medicine and Pharmacy, Cluj-Napoca, Romania; ^5^“RoNeuro” Institute for Neurological Research and Diagnostic, Cluj-Napoca, Romania; ^6^Department of Otorhinolaryngology, Head and Neck Surgery, and School for Oncology and Developmental Biology, Maastricht University Medical Centre, Maastricht, Netherlands; ^7^Department of Health Services Research, Maastricht University, Maastricht, Netherlands

**Keywords:** nutritional status, malnutrition, neurorehabilitation, stroke recovery, stroke rehabilitation, stroke

## Abstract

**Background:**

Malnutrition is common after stroke and can affect rehabilitation and healthcare costs. A comprehensive overview of stroke patients' nutritional condition from the hyperacute to the chronic phase is lacking. This systematic review aimed to investigate the prevalence of impaired nutritional condition (INC) across the continuum of care in specific phases after stroke.

**Methods:**

CAB ABSTRACTS, Embase, MEDLINE, were used to collect studies published between 01-01-1999 and 26-08-2020. Primary and secondary outcomes were prevalence of INC and prevalence of malnutrition, respectively. Exploratory outcomes were prevalence of INC at follow-up, nutritional examination methods, prevalence of dysphagia, stroke severity, adverse events, and continent-specific prevalence of INC. A random-effects meta-analysis model was used to estimate the phase-specific pooled prevalence of INC and malnutrition.

**Results:**

The dataset consisted of 78 study groups selected over a total of 1,244 identified records. The pooled prevalence of INC and malnutrition were 19% (95%CI:7–31) (*N* = 4) and 19% (95%CI:9–29) (*N* = 3), 34% (95%CI:25–43) (*N* = 34) and 26% (95%CI:18–35) (*N* = 29), 52% (95%CI:43–61) (*N* = 34) and 37% (95%CI:28–45) (*N* = 31), 21% (95%CI:12–31) (*N* = 3) and 11% (95%CI:0–24) (*N* = 3) and 72% (95%CI:41–100) (*N* = 3) and 30% (95%CI:0–76) (*N* = 2) in the hyperacute, acute, early subacute, late subacute, and chronic phase, respectively.

**Conclusion:**

INC and malnutrition are highly prevalent in all stages of stroke care. Since malnutrition has been shown to negatively affect clinical outcomes, mortality, and overall healthcare expenditure in stroke survivors, it is essential to examine and monitor the nutritional status of stroke patients throughout their care journey to guide and plan, timely nutritional support and dietary modification.

## Introduction

Malnutrition is common after stroke (1) and relates to poor outcomes as assessed with the modified Ranking Scale, increased prevalence of complications, length of hospital stay, mortality, and hospitalization costs (2, 3). Several factors that occur after stroke, including dysphagia (4), hemiparesis, decreased mobility, depression (5) and post-stroke dementia (6) compound the risk of malnutrition. Multiple studies in stroke patients have consistently demonstrated that the recommended nutritional intake is not achieved after stroke (7–11). Over the past decade, stroke patient outcomes have continuously improved through thrombectomy, recombinant tissue plasminogen activator treatments and case management in stroke units (12). As stroke mortality declines, rehabilitation's importance is growing due to high disability rates among survivors, leading to a high overall burden on global healthcare. In Europe, the total cost of stroke was estimated at €60.0 billion in 2017 (13); almost half of this budget was spent on direct healthcare. The remaining costs were related to informal care, social care systems, non-health or social care areas and productivity losses. Multidisciplinary and structured stroke rehabilitation reduces disability related to stroke regardless of age, sex, and stroke severity (14). Combining neurorehabilitation strategies, such as early mobilization and pharmacological intervention (15, 16), also offers the potential to improve outcomes and reduce costs after stroke. The clinical stroke pathway begins immediately after onset (hyperacute phase), ultimately reaching a chronic phase around six months post-stroke (17). The optimal time window for rehabilitation therapies is considered to be before the observed peak of recovery, between stroke onset and three months after the stroke event (18). Stroke care guidelines recommend using a multidisciplinary approach (5, 14) including nutritional screening and treatment of malnutrition (19, 20). As indicated, malnutrition is common after a stroke. Foley (21) reviewed studies on the prevalence of malnutrition after stroke and possible causes for heterogeneity of its prevalence. They observed a prevalence of malnutrition ranging from 6.1 to 62.0%, but a comprehensive overview of stroke patients' nutritional status from the hyperacute to the chronic phase is lacking. Considering the relevance of nutritional status in the recovery process, this systematic review aimed to investigate the prevalence of impaired nutritional condition (INC), defined as the percentage of not well-nourished patients, across the continuum of care in specific phases after stroke. The term “nutritional condition” is used to describe the results of this review.

## Materials and Methods

### Protocol and Registration

This systematic review was executed following the Preferred Reporting Items for Systematic Reviews and Meta-Analyses (PRISMA) checklist (22) and registered in the international Prospective Register of Systematic Reviews (PROSPERO) (23) (registration number: CRD42020205891).

### Search Strategy

The literature search was performed in ProQuest® by a librarian specialist. ProQuest® was used to inspect three databases (CAB ABSTRACTS, Embase, MEDLINE) for a conceptual string composed of “stroke” (OR synonyms) AND “malnutrition” (OR synonyms). The search was performed for literature published in English between 01-01-1999 to 26-08-2020. Document types excluded from the search were conference abstracts, conference papers, conference reviews, case reports, book chapters, short surveys, retracted publications, letters, editorials, clinical trial protocols, and technical reports. The full search strategy is available in the Supplemental Material (Supplemental Table 1).

### Eligibility Criteria

Meta-analyses, reviews, intended trials, case reports, pharmaceutical clinical trials, and studies including a re-analysis of a study sample were excluded. The population's inclusion criteria were met if the age was ≥ 18 years, and patients were examined for nutritional status within 0 h up to two years after stroke onset. Studies where the nutritional status was used as an eligibility criterion to recruit patients with a specific nutritional status were excluded. Studies were excluded when the entire population was in a comatose/vegetative state or on parenteral nutrition at admission to the study. Selection of the data required that the prevalence of INC was reported in the study as a percentage value or absolute number. The study was excluded if the nutritional status was examined using body mass index (BMI) only. BMI categories might be difficult to interpret considering that both underweight and obese patients can suffer from malnutrition (24). Studies where no indications were provided on the method used for the nutritional examination were excluded. Follow-up data were not included when interventions with an impact on the nutritional status were investigated. If a study reported the prevalence of INC or malnutrition in completely independent groups, the data were treated separately. For example, in studies where the study population was separated into two groups that received the nutritional examination in different time periods, the data on prevalence were treated separately.

### Study Selection and Data Extraction

Duplicates were removed manually. Screening of titles and abstracts was performed by one reviewer (VH or SG). Two reviewers (VH and SG) performed the screening of full-text articles and data extraction for primary and secondary parameters. A third reviewer (CvdB) was consulted in case of a disagreement. One reviewer performed the data extraction of the exploratory parameters (VH), and in case of ambiguity, the second reviewer (SG) was consulted. Percentage values were recalculated for accuracy when needed. Reasons for exclusion of the full-text articles were classified according to the Population, Intervention, Comparison, and Outcome (PICO) framework (25). The PICO framework can be used to systematically identify and document clinical evidence. Studies were excluded if the inclusion criteria related to the “population” (e.g., age) and/or to the “outcome” (e.g., missing prevalence data) were not met, or if there was any other reason for exclusion (e.g., language) which was defined “non-PICO”. The current systematic review does not aim to address research questions related to treatments or differences between intervention and control groups; therefore, the categories “intervention” and “comparison” were not used.

### Outcome Parameters

The primary outcome is the prevalence of INC in each phase after stroke by using the definition of timing described by Bernhardt (17) and limiting the chronic phase to two years after stroke: hyperacute (≤ 24 h), acute (> 24 h– ≤ 7 days), early subacute (> 7 days– <3 months), late subacute (≥ 3 months– <6 months), and chronic (≥ 6 months−2 years). Prevalence of INC at baseline was reported for each study included in the analysis and comprised the full dataset. The secondary outcome is the prevalence of malnutrition in the phases mentioned above. A phase-specific pooled prevalence was estimated for the primary and secondary outcomes. Exploratory outcomes are the prevalence of INC at follow-up, methods used for the nutritional examination (percentage of study groups reporting on screening/assessment tools and anthropometrical/biochemical measurements, and description of the methods), the prevalence of dysphagia, stroke severity evaluated with the National Institutes of Health Stroke Scale (NIHSS), adverse events, and continent-specific prevalence of INC.

### Criteria Used to Estimate the Prevalence of INC and Malnutrition

The prevalence data, as shown in this paper, were based on the method found in the respective study. In case a study reported multiple methods to examine the nutritional status, only one method was selected based on whether it was a method used to generate the primary results or a method largely used in the literature. The methods found in the studies were distinguished in screening/assessment tools or anthropometrical/biochemical measurements. Screening/assessment tools included methods whose outcomes were expressed in pre-defined categories (e.g., no malnutrition, at risk of malnutrition or malnourished). Examples of these tools are, among others, the Malnutrition Universal Screening Tool (“MUST”), and the Mini Nutritional Assessment (MNA). Anthropometrical/biochemical measurements included methods that used measurements of anthropometrical or biochemical parameters. Examples of these measurements are bodyweight and serum albumin levels. The extracted data from the nutritional screening/assessment tools needed to be harmonized according to the definition of INC and malnutrition in this systematic review. There was no need to harmonize data from the Global Leadership Initiative on Malnutrition (GLIM) and the European Society for Clinical Nutrition and Metabolism (ESPEN) diagnostic criteria for malnutrition because in this case the outcomes are not expressed in pre-defined categories but rather on the diagnosis of malnutrition after screening. The criteria used for harmonization of data derived from the remaining screening/assessment tools are shown in the Supplemental Table 2.

### Criteria Used to Estimate the Time of Nutritional Examination After Stroke

In case the time of nutritional examination after stroke (TNE-S-E) was not reported in the study it was estimated according to the following conditions: (i) time of admission after stroke (TA-S) and time of nutritional examination after admission (TNE-A) were available, (ii) TA-S was missing, but information on the phase after stroke was available. The criteria used for the estimation of TNE-S-E are reported in the Supplemental Figure 1.

### Risk of Bias

Risk of bias was evaluated for each study using a self-developed checklist including seven questions related to selection, performance, detection, and reporting bias: (1) Is there a reason to believe that the study population is not representative for the stroke population in the assigned phase after stroke? (selection bias). The answer to this question evaluated whether the setting in which the patients were recruited was representative for the phase to which the study group was assigned. All acute care settings were considered representative for study groups assigned to the hyperacute, or acute phase after stroke. Hospitals, rehabilitation centers, long term care facilities and home (care) were considered representative settings for study groups assigned to the early subacute, late subacute and chronic phase after stroke; however, in case the study was performed in only one of these settings a risk of bias was detected (2) Was the stroke diagnosis confirmed using a CT scan / MRI? (performance bias I) (3) Was a validated screening/assessment tool used for nutritional examination? (performance bias II) (4) Was the method used for the nutritional examination clearly defined in the study? (detection bias I) (5) Was the method used for the nutritional examination consistently used in the study? (detection bias II) (6) Where the prevalence data for all stroke patients who received the nutritional examination available in the study? (reporting bias I) (7) Where the prevalence data complete according to the criteria applied to the screening/assessment tools? (reporting bias II). Question 1, 2, 4, 5, and 6 were scored dichotomously (risk of bias/no risk of bias) and question 3 and 7 were scored trichotomously (risk of bias/no risk of bias/not applicable). Question 3 was not applicable in case the nutritional status was examined using anthropometrical/biochemical measurements. Question 7 was not applicable in case the nutritional status was examined using anthropometrical/biochemical measurements or if GLIM or ESPEN criteria were used. A relative risk of bias [relative risk (%)] was calculated as a percentage of the total number of items that were scored.

### Statistical Analysis

TNE-S-E and TA-S were used as initially reported in the study either as mean (SD), median [range, interquartile range (IQR)] or as a value described in the text. When the mean (SD) was not available, the median (range, IQR) was used. This approach is in line with Hozo (26), who showed that, for sample sizes larger than 25, replacing the sample mean with the reported median is the best estimator for the sample mean. The pooled prevalence of INC and malnutrition was estimated using random-effects (RE) (27) and fixed-effect (FE) (28) meta-analysis models. A RE meta-analysis model was preferred over a FE (29, 30) and used for the interpretation of the results. The between-study variance of the RE model, τ^2^, was estimated via the restricted maximum likelihood approach (31). A meta-analysis of prevalence estimated a weighted average prevalence of the observed proportions, accompanied by a 95% confidence interval (95% CI). NIHSS scores were collected as originally reported in the study, either as a mean or as a median, and used to define the category of stroke severity according to the NIHS Scale. The statistical analyses were carried out in RStudio (R, version 4.0.0; R Project), using the function “rma.uni” from the package *metafor* to pool the raw proportions and package *meta* to create the forest plots.

## Results

**Figure 1:**
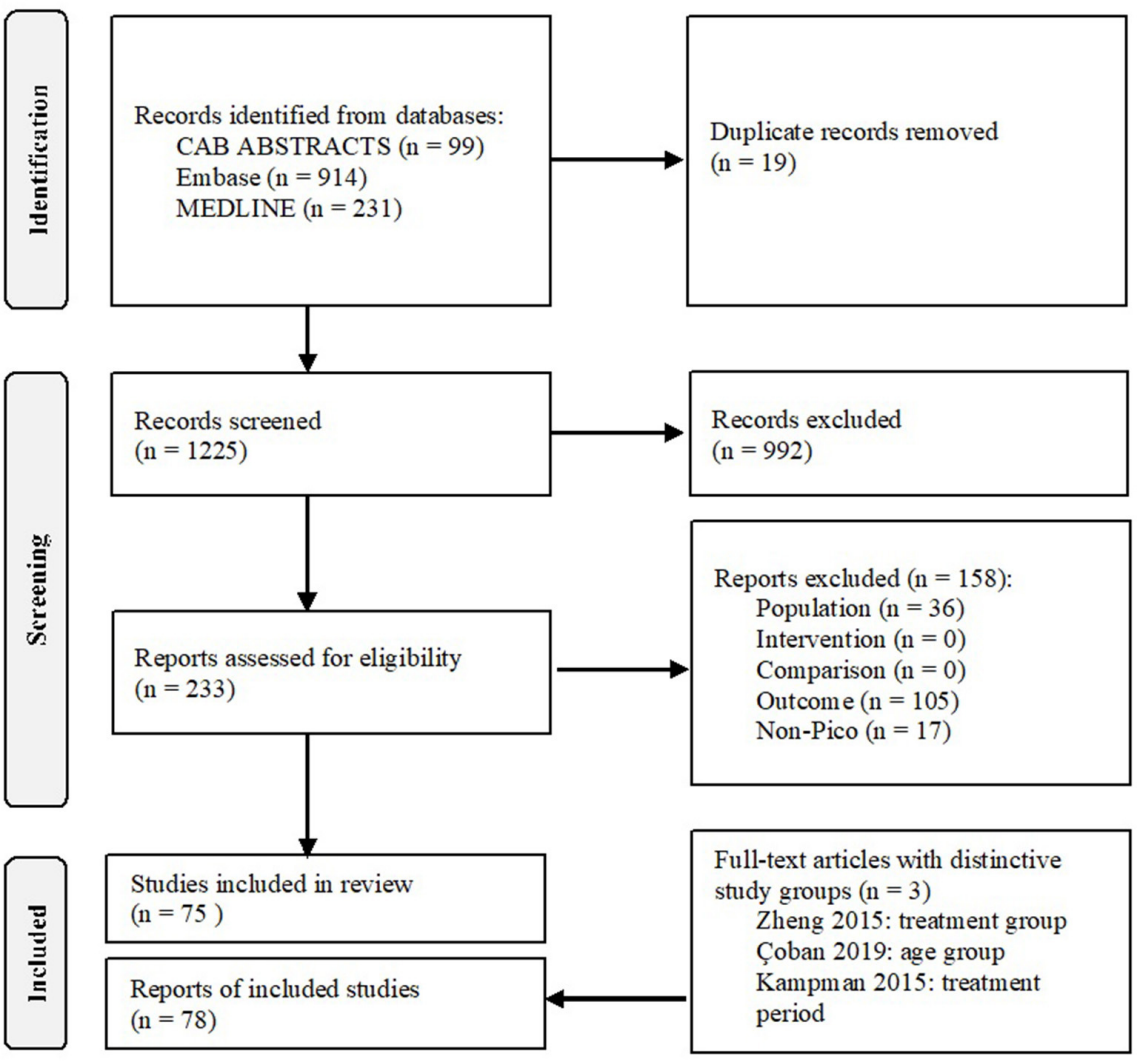
Flowchart. Used under the Creative Commons Attribution License terms, adapted from (22).

A total of 1,244 articles were identified through the literature search, of which 99 in CAB ABSTRACTS, 914 in Embase, and 231 in MEDLINE. A total of 233 full-text articles were assessed for eligibility, of which 75 were included in the analysis. In three studies, the nutritional status was evaluated in independent study groups, and this resulted in a total of 78 study groups (Figure 1).

### General Characteristics of the Study Groups

Study designs were observational and experimental in 68 (87.2%) and 10 (12.8%) study groups, respectively. Fifty-six (71.8%) study groups were performed in hospitals, 17 (21.8%) in rehabilitation facilities, three (3.8%) in nursing homes/care homes/home, and two (2.6%) in a combination of settings. The type of diagnosis reported among the study groups was mainly ischemic and haemorrhagic stroke. TNE-S-E was available and therefore not estimated in 14 (17.9%) out of 78 study groups. The total number of stroke patients who received the nutritional examination was 25,090 ranging from 12 to 4,023 patients per study group. An overview of the general characteristics of the studies is provided in the Supplemental Table 3.

### Prevalence of INC

**Figure 2:**
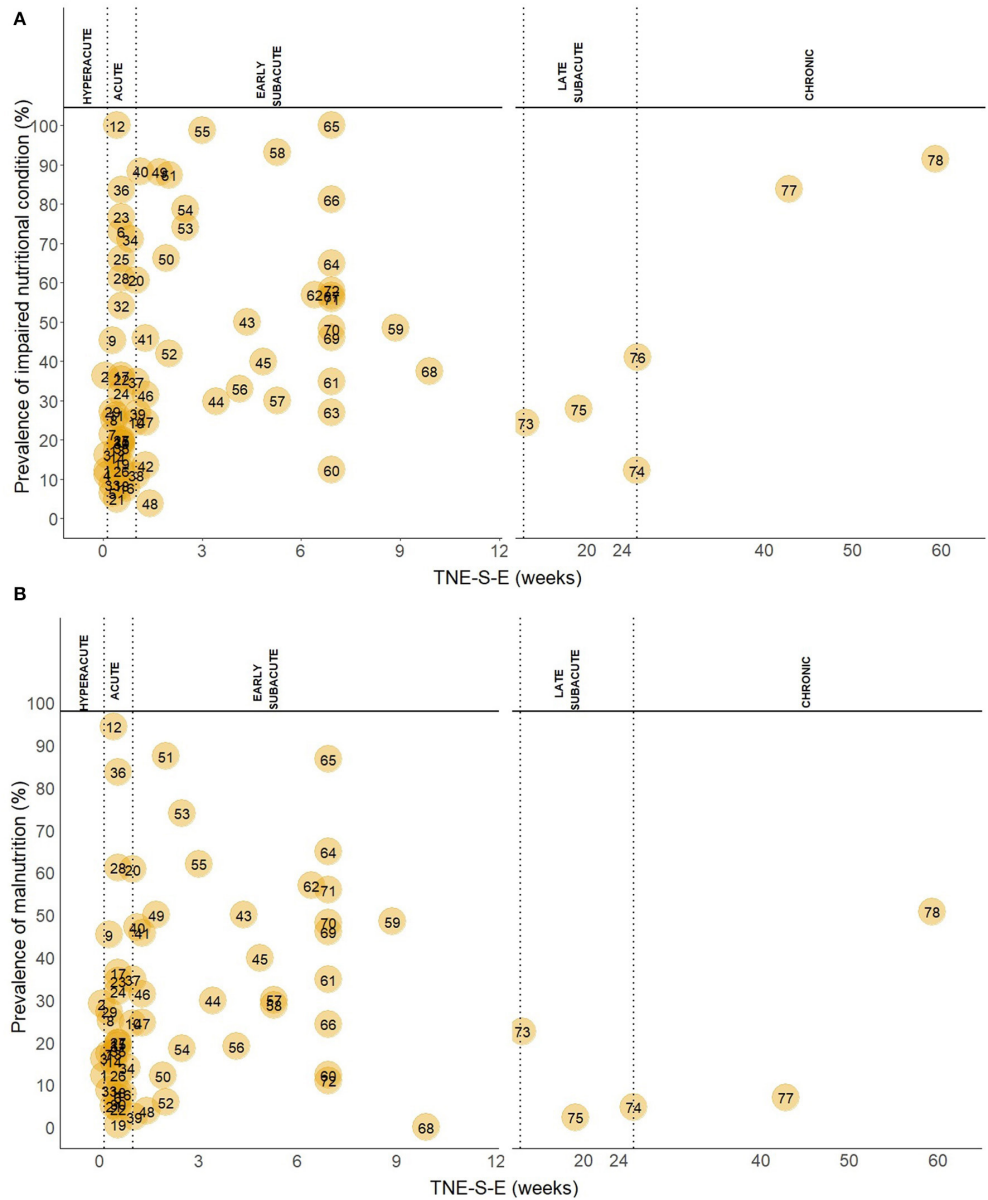
**(A)** Prevalence of INC in the hyperacute, acute, early subacute, late subacute and chronic phase after stroke. TNE-S-E is shown in a different scale in the hyperacute, acute, and early subacute phase compared to the late subacute and chronic phase. Numbers in the plot indicate the references to the study groups and are listed below. **(B)** Prevalence of malnutrition in the hyperacute, acute, early subacute, late subacute and chronic phase after stroke. TNE-S-E is shown in a different scale in the hyperacute, acute, and early subacute phase compared to the late subacute and chronic phase. Numbers in the plot indicate the references to the study groups and are listed: 1, Yoo (2); 2, Gomes (3); 3, Davis (32); 4, Kokura (33); 5, Nozoe (34); 6, Nip (8); 7, Sremanakova (35); 8, Diendéré (36); 9, Vajpayee (37); 10, Gandolfo (38); 11, Crary (39); 12, NanZhu (40); 13, Zheng I (41); 14, Zheng II (41); 15, Shen (42); 16, Food Trial 2005(b) (43); 17, Xiang (44); 18, Kokura (45); 19, Barrio (46); 20, Otsuki (47); 21, Robertson (48); 22, López Espuela (49); 23, Aliasghari (50); 24, Crary (51); 25, Çoban I (52); 26, Çoban II (52); 27, Schwarz (53); 28, Porter (54); 29, Pandian (55); 30, Mosselman (56); 31, Martineau (57); 32, Ha (58); 33, Food Trial 2005(a) (59); 34, Medin (60); 35, Isono (61); 36, Far (62); 37, Brynningsen (63); 38, Kokura (64); 39, Kang (65); 40, Drozdz (66); 41, Cai (67); 42, Naito (68); 43, Hirano (69); 44, Nishioka 2020(b) (70); 45, Nishioka 2020(a) (71); 46, Kampman I (72); 47, Kampman II (72); 48, Zhang (73); 49, Shiraishi (74); 50, Hsieh (75); 51, Falsetti (76); 52, Sato (77); 53, Lim (78); 54, James (79); 55, Nishioka (80); 56, Aadal (81); 57, Aquilani (10); 58, Nishioka (82); 59, Garbagnati (83); 60, Westergren (84); 61, Poels (85); 62, Hama (86); 63, Maruyama (87); 64, Shimizu (88); 65, Carlsson (89); 66, Tsai (90); 67, Kaur (91); 68, Jung (92); 69, van Zwienen-Pot (93); 70, Campillo (94); 71, Da Silva (95); 72, Lelli (96); 73, Scrutinio (97); 74, Perry (11); 75, Vilardell (98); 76, Westergren (99); 77, Choi (100); 78, Kim (101).

**Figure 3:**
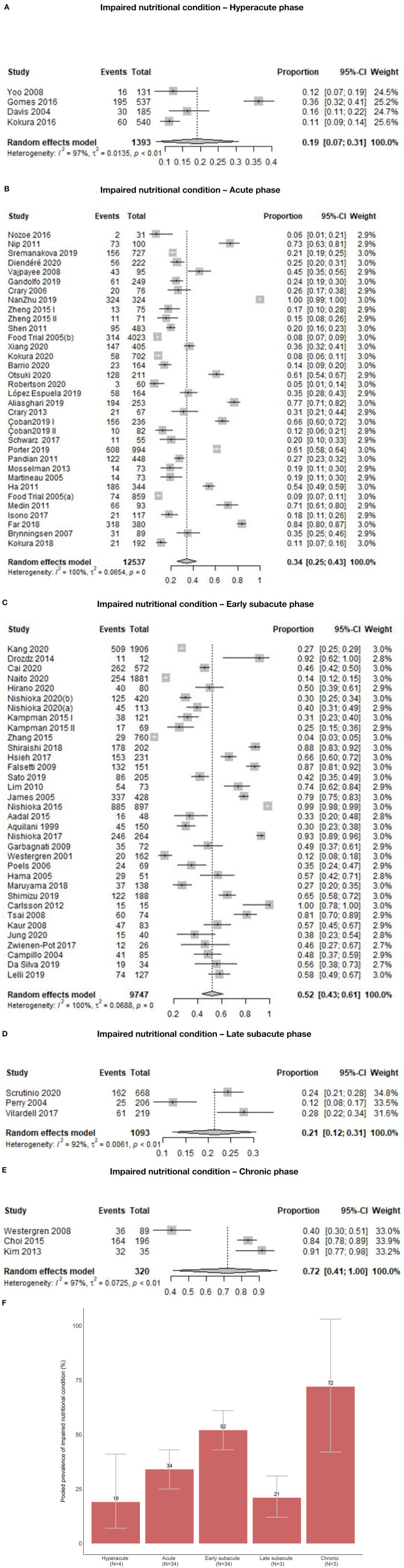
**(A)** Pooled prevalence of INC in the hyperacute phase. **(B)** Pooled prevalence of INC in the acute phase. **(C)** Pooled prevalence of INC in the early subacute phase. **(D)** Pooled prevalence of INC in the late subacute phase. **(E)** Pooled prevalence of INC in the chronic phase. **(F)** Pooled prevalence of INC per phase.

Out of 78 study groups with data on INC, four (5.1%) were conducted in the hyperacute, 34 (43.6%) in the acute, 34 (43.6%) in the early subacute, three (3.8%) in the late subacute, and three (3.8%) in the chronic phase. Overall, the prevalence of INC across phases ranged from 3.8 to 100.0%. Prevalence of INC ranged from 11.1 to 36.3% in the hyperacute phase, 5.0 to 100% in the acute phase, 3.8 to 100% in the early subacute phase, 12.1 to 27.8% in the late subacute phase and 41 to 91.4% in the chronic phase (Figure 2A). Combining the individual prevalence numbers per phase yielded a pooled prevalence of 19% (95%CI: 7–31) based on four study groups in the hyperacute phase, 34% (95%CI: 25–43) based on 34 study groups in the acute phase, 52% (95%CI: 43–61) based on 34 study groups in the early subacute phase, 21% (95%CI: 12–31) based on three study groups in the late subacute phase, and 72% (95%CI: 41–100) based on three study groups in the chronic phase (Figure 3). In the phases where the pooled prevalence was based on a number of study groups ≤ 5, the results generated with the RE and FE meta-analysis models were overall similar.

### Prevalence of Malnutrition

**Figure 4:**
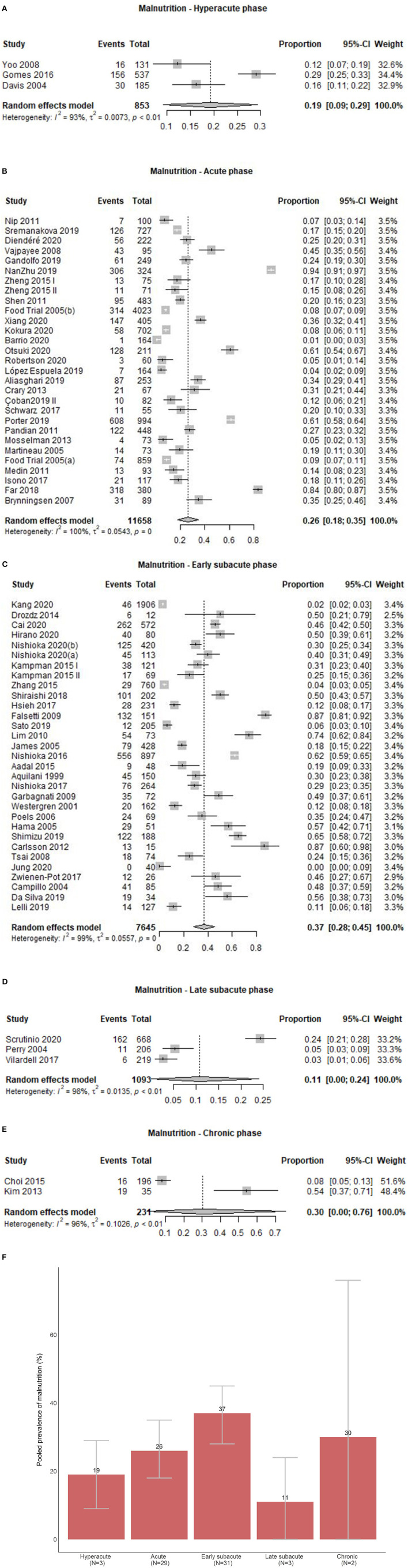
**(A)** Pooled prevalence of malnutrition in the hyperacute phase. **(B)** Pooled prevalence of malnutrition in the acute phase. **(C)** Pooled prevalence of malnutrition in the early subacute phase. **(D)** Pooled prevalence of malnutrition in the late subacute phase. (**E**) Pooled prevalence of malnutrition in the chronic phase. **(F)** Pooled prevalence estimates of malnutrition per phase.

Of 68 study groups with data on malnutrition, three (4.4%) were conducted in the hyperacute, 29 (42.6%) in the acute, 31 (45.6%) in the early subacute, three (4.4%) in the late subacute, and two (2.9%) in the chronic phase after stroke. Overall, the prevalence of malnutrition across phases ranged from 0.0 to 94.4%. Prevalence of malnutrition ranged from 12.2 to 29.1% in the hyperacute phase, 0.6 to 94.4% in the acute phase, 0.0 to 87.4% in the early subacute phase, 2.7 to 24.3% in the late subacute phase, and 7.7 to 54.3% in the chronic phase (Figure 2B). Combining the individual prevalence numbers per phase yielded a pooled prevalence of 19% (95%CI:9–29) based on three study groups in the hyperacute phase, 26% (95%CI:18–35) based on 29 study groups in the acute phase, 37% (95%CI:28–45) based on 31 study groups in the early subacute phase, 11% (95%CI:0–24) based on three study groups in the late subacute phase, and 30% (95%CI:0–76) based on two study groups in the chronic phase (Figure 4). In the phases where the pooled prevalence was based on a number of study groups ≤ 5, the results generated with the RE and FE meta-analysis models were overall similar, except for the chronic phase where the FE meta-analysis model showed a pooled prevalence of 10%.

### Prevalence of INC at Follow-Up

**Figure 5:**
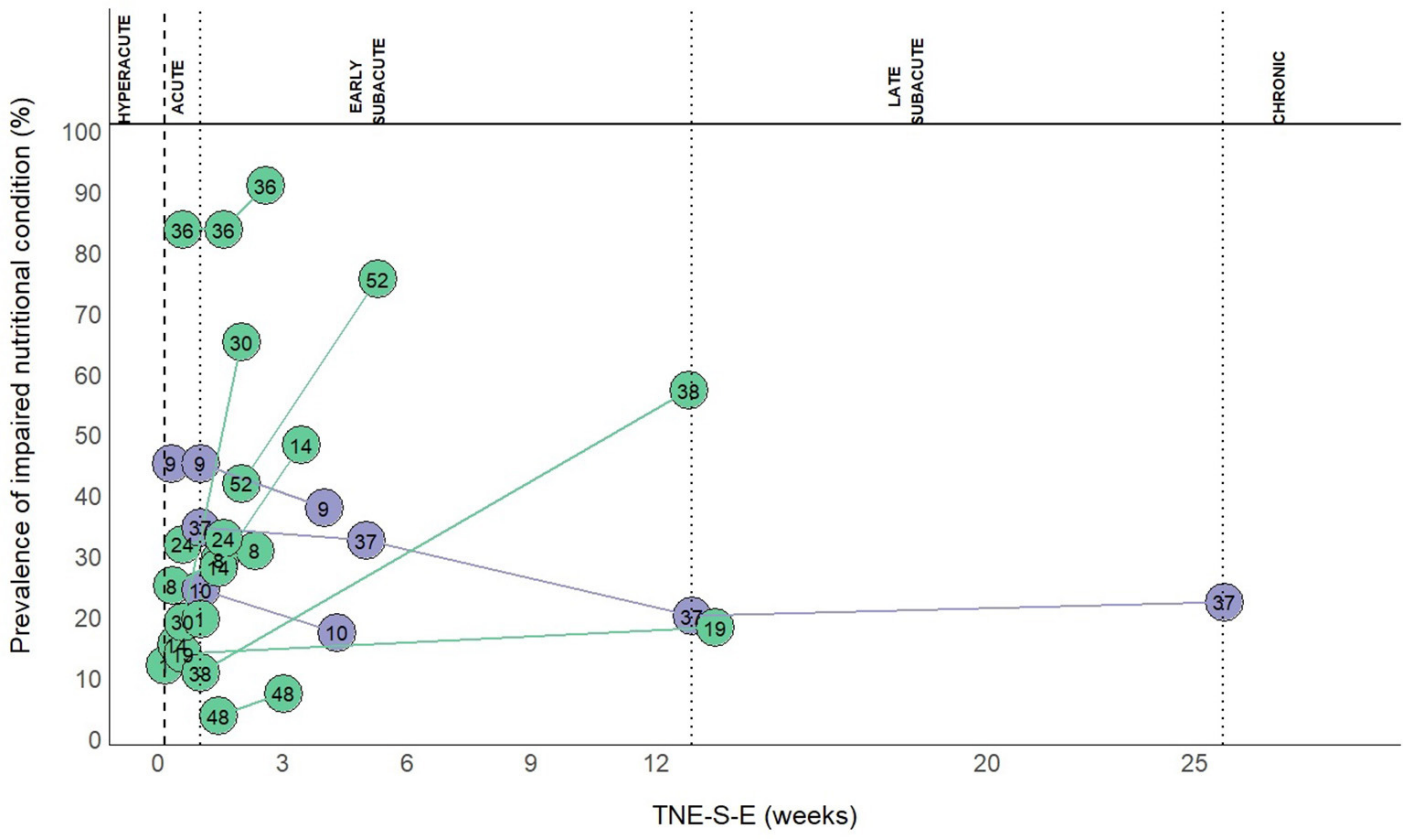
Prevalence of INC at follow-up. Numbers in the plot indicate the references to the study groups: 1, Yoo 2008 (2); 8, Diendéré 2020 (36); 9, Vajpayee 2008 (37); 10, Gandolfo 2019 (38); 14, Zheng 2015 II (41); 19, Barrio 2020 (46); 24, Crary 2013 (51); 30, Mosselman 2013 (56); 36, Far 2018 (62); 37, Brynningsen 2007 (63); 38, Kokura 2018 (64); 48, Zhang 2015 (73); 52, Sato 2019 (77).

Follow-up data on INC at different time points were available in 13 (16.7%) out of the 78 study groups. An increased prevalence of INC occurred in most of these 13 study groups and within three months after stroke (Figure 5).

### Methods Used for the Nutritional Examination

**Figure 6:**
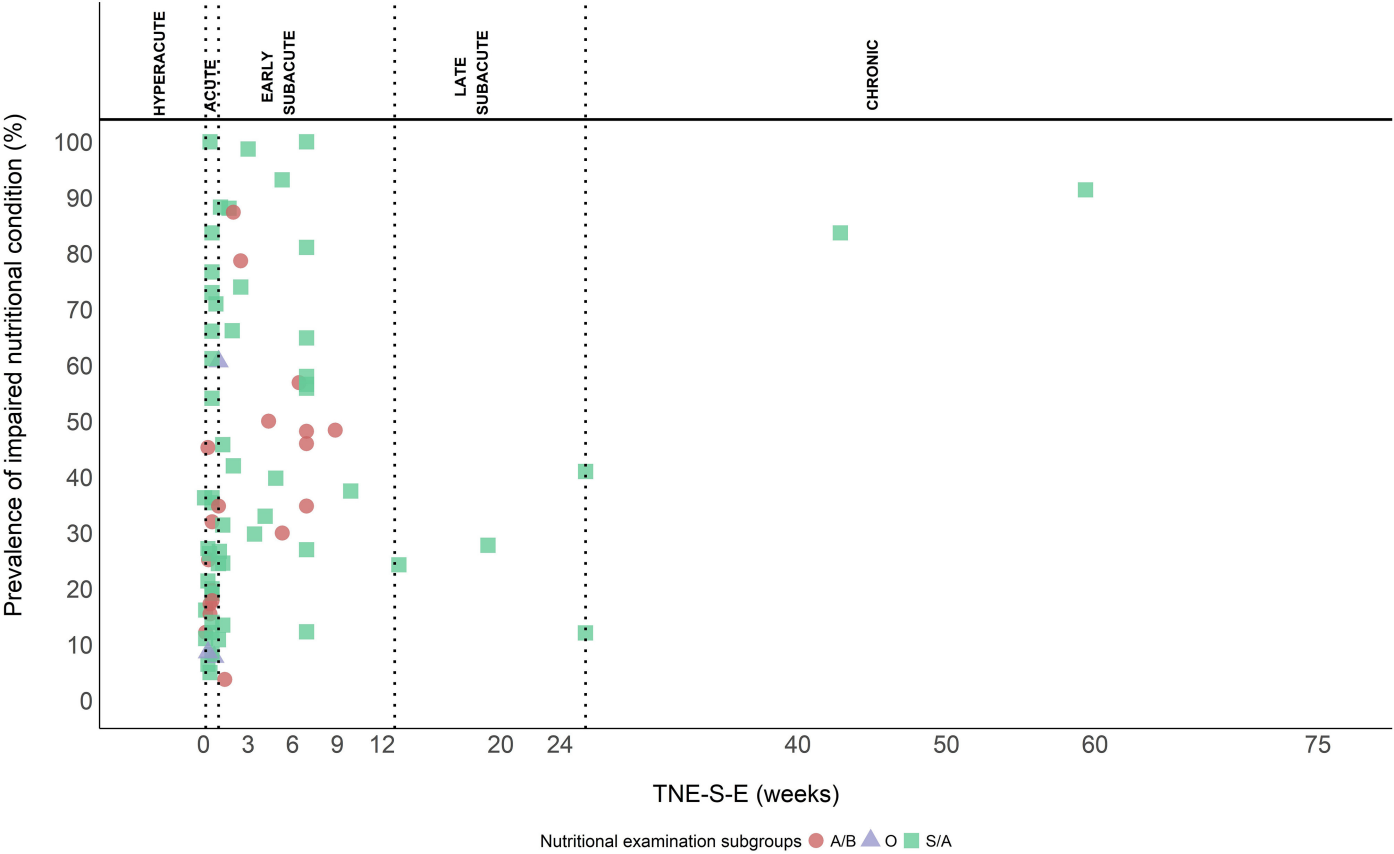
Methods used to examine the prevalence of INC. Screening/assessment tools (S/A) (squares), anthropometrical/biochemical measurements (A/B) (circles), and other (O) (triangles) (combination of S/A tools and A/B measurements).

Screening/assessment tools and anthropometrical/biochemical measurements were used for the nutritional examination in 56 (71.8%), and 19 (24.4%) out of the 78 study groups, respectively, and three (3.8%) reported various methods. Twenty (35.7%) of the 56 study groups used the MNA (102) or the MNA short-form (MNA-sf) (103), eight (14.3%) used the Geriatric Nutritional Risk Index (GNRI) (104), seven (12.5%) used the Subjective Global Assessment (SGA) (105), seven (12.5%) used the Nutrition Risk Score (NRS) (106), four (7.1%) used the “MUST” (107), three (5.4%) used the Patient-generated Subjective Global Assessment (PG-SGA) (108), two (3.6%) used the Prognostic Nutritional Index (PNI) (109, 110), two (3.6%) used the ESPEN diagnostic criteria for malnutrition (111), one (1.8%) used the Malnutrition Screening Tool (MST) (112), one (1.8%) used the Controlling Nutritional Status score (CONUT) (113), and one (1.8%) used the GLIM criteria (114). Out of the 19 study groups evaluating the nutritional status with anthropometrical/biochemical measurements, nine (47.4%) used a combination of anthropometrical and biochemical measurements, four (21.1%) used anthropometrical measurements, and six (31.6%) used biochemical measurements only. Anthropometrical measurements included BMI, bodyweight (loss), weight index based on actual bodyweight and reference weight (2, 115), arm muscle circumference, triceps skinfold, and the brachial perimeter. Biochemical measurements included albumin, pre-albumin, transferrin, hemoglobin, total cholesterol, lymphocyte count, ferritin, transthyretin, iron, and urea. In three study groups, a combination of screening/assessment tools and anthropometrical/biochemical measurements was used, and it included either a combination of bedside assessment, bodyweight, height, dietary history, blood test, or “MUST” and albumin. Figure 6 shows the prevalence of INC examined with different methods and plotted against TNE-S-E.

### Prevalence of Dysphagia, Stroke Severity, Adverse Events, and Continent-Specific Prevalence of INC

Thirty-two (41.0%) of the 78 study groups reported on the prevalence of dysphagia at baseline in the stroke patients in whom nutritional status was examined. After excluding study groups that used the presence or absence of dysphagia as an eligibility criterion, the prevalence of dysphagia ranged between 6.0 and 87.5%. A wide variety of screening and diagnostic methods were used to assess dysphagia. Out of the 78 study groups, 20 (25.6%) reported NIHSS scores at baseline in the stroke patients who were examined for the nutritional status. Mean/median NIHSS scores ranged from 1.5 to 14.2. Minor (NIHSS scores 1–4) and moderate (NIHSS scores 5–15) strokes were reported in three (15.0%) and 17 (85.0%) of the 20 study groups, respectively. Poor nutritional status was often linked to adverse events such as post-stroke complications and poor outcomes. Studies reported pressure ulcer development, impaired functional independence, a longer length of hospital stay, hospitalization costs, unfavorable recovery from stroke, and increased mortality. The continent-specific pooled prevalence of INC was 46% (95%CI: 36–56) based on 36 (46.2%) study groups in Asia, 37% (95%CI: 28–45) based on 29 (37.2%) study groups in Europe, 36% (95%CI: 16–56) based on 7 (9.0%) study groups in Australia, 46% (95%CI: 13–79) based on 3 (3.8%) study groups in North-America, 74% (95%CI: 39–100) based on two (2.6%) study groups in South-America, 25% (95%CI: 30–31) based on one (1.3%) study group in Africa, and 42% (95%CI: 36–48) based on the total number of 78 study groups (Supplemental Figure 2). In the continents where the pooled prevalence was based on a number of study groups ≤ 5, the results generated with the RE and FE meta-analysis models were overall similar.

### Risk of Bias

**Figure 7:**
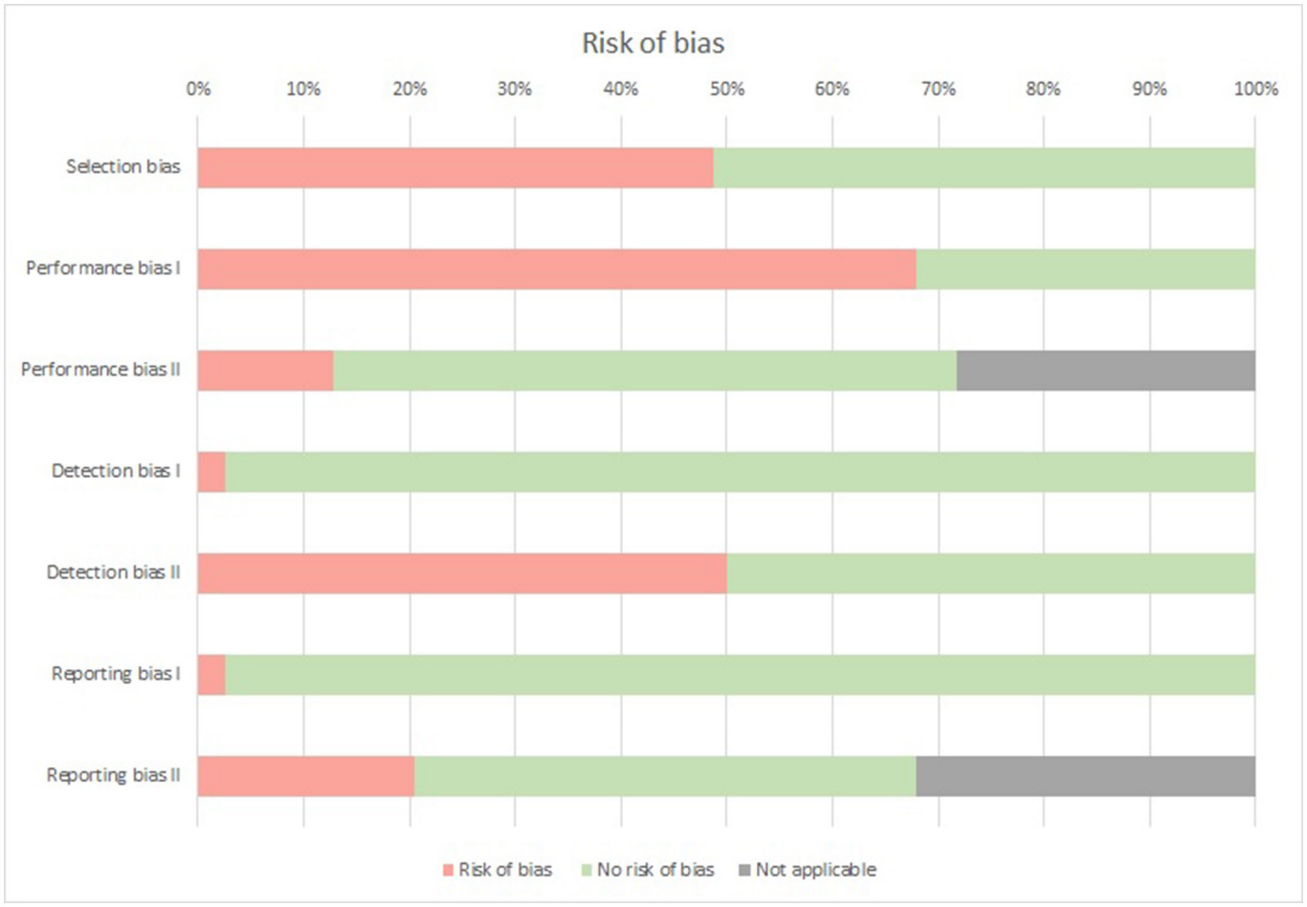
Risk of bias summary.

A risk of selection bias was found in 38 out of the 78 study groups (48.7%) as the study population was considered not representative for the stroke population in the assigned phase after stroke. A risk of performance bias was found in 53 out of 78 (67.9%) study groups based on methods used for the confirmation of stroke (performance bias I). In these study groups this information was unknown, not reported, or the diagnosis was confirmed differently, e.g., screening by a board certificated neurologists or extraction of data from the patients' medical dossiers. A risk of performance bias based on validity of the screening/assessment tools for nutritional examination (performance bias II) was found in 10 out of the 56 (17.9%) study groups that used screening/assessment tools for the examination of nutritional status. These study groups used e.g., the GNRI or PNI, that have not been validated in specific patient populations. A risk of detection bias was found in two out of 78 (2.6%) study groups as these did not clearly define the methods used to examine the nutritional status (detection bias I). A risk of detection bias was also found in 39 out of 78 (50.0%) study groups as there was no clear indication of consistent performance of methods (detection bias II). In these study groups, it was unclear who performed the evaluation or who collected the data from medical files or a wide variety of assessors was involved. A risk of reporting bias was found in two of the 78 (2.6%) study groups as these study groups included about 99% of confirmed stroke and remaining subjects were diagnosed with “brain tumor” or as “non-stroke” (reporting bias I). In 16 out of 53 (30.2%) study groups that used screening/assessment tools other than GLIM or ESPEN criteria for the examination of nutritional status, reported incomplete prevalence data on INC according to the criteria (Supplemental Table 2) used in this systematic review (reporting bias II). In these cases, data were missing in one or more categories. A summary of the risk of bias is provided in Figure 7. The risk of bias and relative risk for each individual study group is provided in the Supplemental Table 4.

## Discussion

This systematic review shows the prevalence of INC and malnutrition ranging from 3.8 to 100.0% and from 0.0 to 94.4%, respectively. A high prevalence of INC was reported within three months after stroke. The pooled prevalence of INC was 34% in the acute and 52% in the early subacute phase, respectively. For malnutrition, these numbers were 26 and 37%, respectively. A deterioration of nutritional condition within the first three months was seen from the follow-up data. A poor nutritional condition occurring within three months after stroke parallels the time period associated with the peak of recovery (17, 18). As poor nutritional status negatively impacts the recovery processes, it is advised to intervene within this time window and to address nutrition as an integral component of rehabilitation therapy.

The importance of nutrition in stroke recovery is supported by several studies that demonstrate an association between poor nutritional status and worse stroke outcomes such as disability, complications, extended length of hospital stay, mortality and costs for hospitalization (2, 3). Poor nutritional status, inactivity and immobilization, can lead to muscle loss and sarcopenia and can negatively impact the recovery after stroke (116). A recent meta-analysis by Su (117) reports that sarcopenia is common after stroke. Furthermore, several studies show that improving the nutritional status of stroke patients using specialized nutritional interventions can significantly improve clinical outcomes. In a randomized controlled trial (RCT) with 102 undernourished stroke patients, intensive nutritional supplementation, including oral nutritional supplements (ONS), improved motor function (*p* < 0.002) (118). In a rehabilitation center the total Functional Independence Measure (FIM) gain (*p* = 0.036) and efficiency (*p* = 0.020) were improved in cerebrovascular patients (mainly due to stroke) with poor nutritional status and in whom an improvement of the GNRI and energy intake was achieved (119). A different RCT showed that supplementation of subacute ischemic stroke patients with high protein ONS enhanced the cognitive function evaluated with the Mini-Mental State Examination (*p* = 0.01) (120). Oral energy and protein-rich (enteral) feeding of acute stroke patients at nutritional risk increased quality of life (*p* = 0.009) and handgrip strength (*p* = 0.002) (121). A positive effect on energy (*p* < 0.0001) and protein (*p* < 0.001) intake and on albumin (*p* = 0.025) and iron (*p* = 0.030) levels were observed in acute ischemic stroke patients using ONS providing 600 Kcal and 20 g protein per day in addition to the hospital diet compared to stroke patients randomized to receive only the hospital diet (122). A recent study investigated the effect of tailored dietary prescription in 454 stroke patients in rehabilitation and reported an inverse correlation between dysphagia and frequency of dietary adjustments in prescriptions (*p* = 0.032) (123). In addition, more frequent dietary adjustments positively affected FIM motor scores (*p* = 0.045), muscle mass change (*p* = 0.028), and length of hospitalization (*p* = 0.019) (123). The Feed Or Ordinary Diet (FOOD) trial randomized acute non-dysphagic stroke patients to a control group that received a regular hospital diet or a treatment group that received a regular hospital diet with additional ONS that did not measurably affect mortality or outcome (43). However, 77.0% of the population in the FOOD Trial was well-nourished at baseline, and this may have influenced the effectiveness of ONS. Finally, the importance of examination of nutritional status and dysphagia and adequate nutritional status in stroke patients is reflected in several (international) stroke guidelines. These guidelines recommend dysphagia screening prior to first oral intake in all stroke patients, screening for malnutrition and the provision of nutritional support, including the use of ONS, in stroke patients with an impaired nutritional status and/or dysphagia (1, 19, 20, 124–127). These guidelines (1, 19, 126, 127) do not recommend routine administration of ONS in well-nourished stroke patients, in line with the results of the FOOD trial. In these guidelines, also recommendations are given on the use of enteral tube feeding in specific conditions and/or on the route of administration (nasogastric or PEG) (19, 20, 127).

This review shows variation in the prevalence of INC. This may be attributed to the various methods used to screen or assess the nutritional status. A gold standard method and a recognized definition of malnutrition are lacking (128). Only recently, the GLIM reached a global consensus on the diagnostic criteria for malnutrition in adults. Nutritional screening and assessment are both included, and five key health phenotypic and etiologic health criteria such as involuntary weight loss, BMI, decreased muscle mass, reduced nutritional intake or absorption, and disease-induced burden or inflammation are covered (114). Foley (21) suggested that a great part of the variation in the estimates of malnutrition in stroke may be attributed to differences in the nutritional examinations. In this systematic review, 71.8% of the study groups used screening/assessment tools and 24.4% of the study groups used anthropometrical/biochemical measurements. The results showed a higher prevalence of INC when the screening/assessment tools were used, indicating that the prevalence may vary in relation to the methods used for the examination. Additionally, in some cases, the original tools were modified, and the adapted versions were used for the examination. The use of one single method might result in significant prevalence variations as well. Geriatric patients showed a prevalence of malnutrition and risk of malnutrition between 3.0 and 58.0% when the nutritional examination was performed with MNA solely (129). Considerable variation of the prevalence of malnutrition was also observed within patient groups. In cancer patients, the type of cancer was an important determinant (130, 131). In addition, the setting in which patients are residing could also play a role. Cereda (132) reported on the nutritional status in older people examined with MNA in various settings. They found high heterogeneity in the studies, with a prevalence of malnutrition ranging between 3.1% in the community and 29.4% in rehabilitation/post-acute care. Studies in the current review have also been performed in a variety of health care settings. The time of nutritional examination has been suggested to be a reason for the variation of prevalence in stroke patients as well (21). Although in this systematic review, the timing was taken into account by studying each phase after a stroke, a considerable variation of the prevalence remained. The Stroke Recovery and Rehabilitation Roundtable Taskforce (17) encourages research in the field to provide clear guidance on timing. TNE-S-E was only available in 17.9% of the study groups; the allocation of the remaining studies within a pre-defined phase provides a general indication of the time of nutritional examination after a stroke. The studies included in the analysis were not all explicitly performed to examine the nutritional status in stroke, and this might have contributed to the missing data on timing.

When interpreting the data on the prevalence of INC in the hyperacute phase, it is crucial to consider the limited time passed since stroke onset. Data on nutritional status in this phase most likely indicate the state of nutrition before the stroke event rather than an actual stroke-related change in nutritional status. However, some screening/assessment tools determine nutritional risk by allocating a score to reduced or interrupted nutritional intake which would reflect that moment in time. The small number of studies reporting on the nutritional status in the hyperacute phase is likely a result of the significant focus on specific treatments and patient needs within 24 h after stroke. Lack of data in the late subacute and chronic phase might reflect a reduced number of studies performed at later stages or a lack of attention to the nutritional status over time. Considering the high prevalence of INC occurring within three months after stroke, continuous monitoring of the nutritional status during and beyond this stage of rehabilitation is desirable. The current review shows the prevalence of dysphagia up to 87.5%, and Foley (133) reports that dysphagia increases the risk of malnutrition 2.4 fold (*p* < 0.008). This systematic review highlights the need for future research to increase the knowledge on nutritional status after stroke.

To our knowledge, this systematic review has been performed in the most appropriate way to provide a transparent and comprehensive overview of the existing evidence. Nevertheless, this study has some limitations. The screening of titles and abstracts and data extraction of the exploratory parameters was performed by one reviewer, and data on prevalence and timing were harmonized with specific self-developed criteria. One other limitation is that screening and assessment tools were both included, and a differentiation (134) was not performed. Despite this limitation, all eligible literature on nutritional status in stroke was considered valuable and included in the analysis of the review. The risk of bias was evaluated using a self-developed checklist. This checklist included critical questions regarding selection, performance, detection, and reporting bias and provided a comprehensive risk of bias summary. Due to the high heterogeneity of the data one may not conclude on the exact prevalence of INC; however these results shed light on a problem that is often underestimated.

## Summary

In summary, results of the current review indicate that INC and malnutrition occur across the continuum of stroke care, from the hyperacute to the chronic phase. The large prevalence range of INC and malnutrition in the different phases underlines the importance of continuously reviewing the nutritional status in stroke patients to identify and take action to prevent nutritional deterioration. The large prevalence range also shows that there is a large heterogeneity in prevalence data amongst different studies. Malnutrition after stroke has been shown to negatively affect clinical outcomes, mortality and overall healthcare expenditure. This suggests that continuous monitoring of the nutritional status and improved nutritional management within the multidisciplinary context of rehabilitation is warranted, to ensure malnutrition does not go unnoticed, untreated, and impede rehabilitation and recovery after stroke.

## Data Availability Statement

The original contributions presented in the study are included in the article/Supplementary Material, further inquiries can be directed to the corresponding author.

## Author Contributions

SG and ML initiated the study. SG and VH performed the screening of the literature and the data extraction. All authors contributed to the data analysis, review, interpretation of the results, were involved in the writing of the manuscript, and provided their consent on the manuscript.

## Funding

Support for this work has been received from Maastricht University, Maastricht, Netherlands and from Danone Nutricia Research, Utrecht, Netherlands.

## Conflict of Interest

SG, ML, and AHe are employees of Danone Nutricia Research. JS and AHo have been consultants for Danone Nutricia Research. VH and JS received financial support for their research. LB is a consultant for Phagenesis Limited, Manchester, United Kingdom. The authors declare that this study received funding from Danone Nutricia Research. The authors employed by the funder had co-involvement in the following parts of the study: design, data collection, analysis, interpretation of data, the writing of this article and the decision to submit it for publication.

## Publisher's Note

All claims expressed in this article are solely those of the authors and do not necessarily represent those of their affiliated organizations, or those of the publisher, the editors and the reviewers. Any product that may be evaluated in this article, or claim that may be made by its manufacturer, is not guaranteed or endorsed by the publisher.
