# Supplementary material for: Impaired Nutritional Condition After Stroke From the Hyperacute to the Chronic Phase: A Systematic Review and Meta-Analysis
Source: Front Neurol. 2022 Feb 1;12:780080. doi: 10.3389/fneur.2021.780080 (PMC8846185; doi:10.3389/fneur.2021.780080)
Supplement: Supplementary file 1 [file Data_Sheet_1.pdf]

SUPPLEMENTARY INFORMATION

Table S1. Search strategy

Conceptual String:	Ti,ab(stroke) AND Ti,ab(malnutrition)
String:	Ti,Ab((((cerebrovascular OR cerebral OR brain) n/2 (trauma OR traumas OR injur* OR occlusion OR occlusions OR haemorrhage OR haemorrhages OR hemorrhage OR hemorrhages OR accident OR accidents OR lesion OR lesions OR vasculopathy OR attack OR attacks OR isch*emia OR infarct* OR insult OR insults OR reperfusion OR CVA)) OR (stroke OR strokes))) AND Ti,ab(malnutrition OR malnourish* OR (nutrition* p/0 (status OR intake)) OR undernourish* OR underweight OR (low p/0 body p/0 (mass OR weight)) OR underfed OR underfeeding OR undernutrition OR (nutrition* p/0 (depriv* OR deficien*)))
Databases:	CAB ABSTRACTS, Embase, MEDLINE
Publication date:	Between 01-01-1999 and 26-08-2020
Language	English
Document type exclusions	(Conference Abstract AND Case Reports AND Conference Paper AND Conference paper AND Book chapter AND Short Survey AND Conference Review AND Retracted Publication AND Letter AND Editorial AND Note AND Book AND Chapter AND Comment AND Published Erratum AND Bulletin article AND Clinical Trial Protocol AND Erratum AND Tombstone AND Address AND Technical Report)
**# of results:**	**1244**

Table S2. Criteria used to estimate the prevalence of INC and malnutrition when examined with screening/assessment tools.

		INC	Malnutrition
**Malnourished and at risk of malnutrition***			
**MNA**	no malnutrition (24-30)		
	at risk of malnutrition (17-23.5)	✓	
	malnourished (<17)	✓	✓
**MNA-sf**	no malnutrition (12-14)		
	at risk of malnutrition (8-11)	✓	
	malnourished (0-7)	✓	✓
**At risk of malnutrition****			
**MUST**	low risk of malnutrition (0)		
	medium risk of malnutrition (1)	✓	
	high risk of malnutrition (2)	✓	✓
**GNRI**	no risk of malnutrition (>98)		
	low risk of malnutrition (92-98)	✓	
	moderate risk of malnutrition (82-92)	✓	
	severe risk of malnutrition (<82)	✓	✓
**NRS**	no risk of malnutrition (0)		
	mild risk of malnutrition (1)	✓	
	moderate risk of malnutrition (2)	✓	
	severe risk of malnutrition (≥3)	✓	✓
**MST**	no risk of malnutrition (0-1)		
	at risk of malnutrition (≥2)	✓	✓
**Severity of malnutrition*****			
**SGA**	no malnutrition (A)		
	moderate/suspected malnutrition (B)	✓	✓
	severe malnutrition (C)	✓	✓
**PG-SGA**	no malnutrition (A) (0-1)		
	moderate/suspected malnutrition (B)	✓	✓
	severe malnutrition (C) (≥9)	✓	✓
**CONUT**	no malnutrition (0-1)		
	mild malnutrition (2-4)	✓	✓
	moderate/severe malnutrition (≥5)	✓	✓
**PNI**	no malnutrition (≥ 50)		
	mild malnutrition (< 50)	✓	✓
	moderate/severe malnutrition (< 45)	✓	✓
	serious malnutrition (< 40)	✓	✓
**Malnutrition based on multiple criteria******			
**ESPEN criteria**	Validated screening tool, BMI, weight loss, fat-free mass index	malnutrition	malnutrition
**GLIM criteria**	Validated screening tool, phenotypic and etiologic criteria	malnutrition	malnutrition

Table S2. Legend

* The prevalence of INC was defined as the combination of the categories “malnourished” and “at risk of malnutrition” and the prevalence of malnutrition was defined by the category “malnutrition”.

****** The prevalence of INC was defined as the combination of the categories “medium risk” and “high risk” and the prevalence of malnutrition was defined by the most severe category (“high risk”). The most severe category was used to estimate malnutrition based on the principle that patients who are at high nutritional risk need to be treated, as indicated in the management guidelines of the “MUST”.

*** The prevalence of INC and malnutrition were both defined by all the categories of malnutrition. The reason for not differentiating between INC and malnutrition is that in some cases a specific description of the nutritional status was not provided as shown by one of the pre-defined categories of the SGA or PG-SGA that combines both “moderate” and “suspected” malnutrition.

**** The use of pre-defined outcome categories was not applied. Malnutrition was defined based on multiple criteria, and it was not possible to differentiate INC and malnutrition.

Figure S1. Criteria used to estimate TNE-S-E

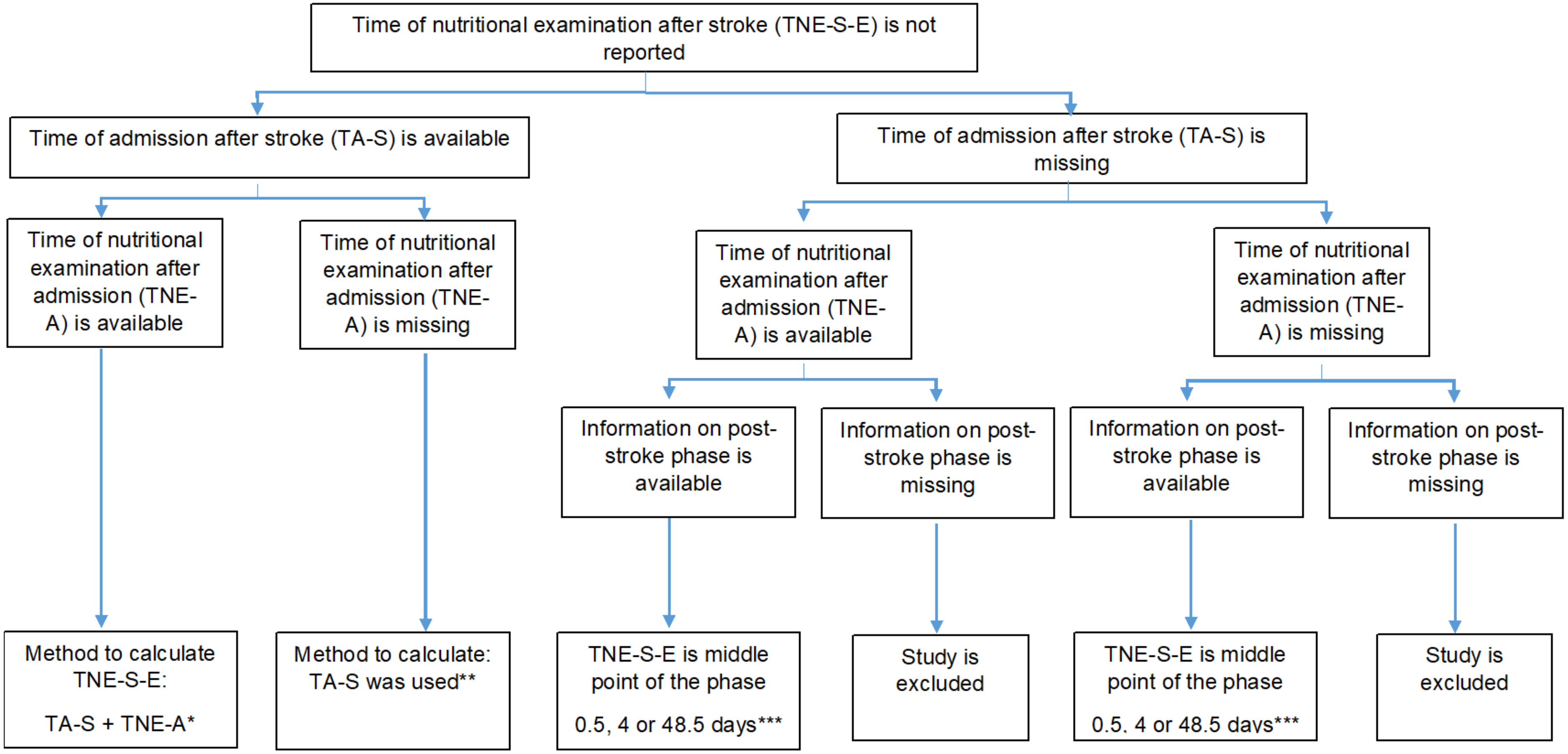

Figure S1. Legend

* TNE-S-E was estimated using TA-S and TNE-A. For example, in a study where stroke patients were admitted within three days after stroke (TA-S) and nutritional examination was performed within seven days after admission (TNE-A), the TNE-S-E was ten days. TNE-A does not indicate timing after stroke; therefore, it did not affect the estimations except when used combined with TA-S.

** Estimation of TNE-S-E was not needed if TA-S was available and TNE-A was missing. TA-S was considered equal to TNE-S-E since it was assumed that the nutritional examination was performed soon after the admission.

*** TA-S was missing, but information on the phase was available. In this case, the middle point of the phase was used as guidance: for the hyperacute, acute, and early subacute phase, TNE-S-E was assumed at 0.5, 4, and 48.5 days, respectively. If the study was performed in a rehabilitation setting, it was assumed that the patients were in the early subacute and TNE-S-E was assumed at 48.5 days. If the study was performed with chronic stroke patients, TNE-S-E was not estimated to prevent the inclusion of patients at more than two years after stroke.

In two studies, the criteria used to estimate the time of nutritional examination after stroke were not applicable because the estimations deviated compared to the data reported in the original studies. In both studies only the TNE-A and the indication of the phase were available. According to the criteria, the time of nutritional examination after stroke was 4 (47) and 48 (66) days based on the acute and early subacute phase, respectively. Otsuki (47) reports that the nutritional examination was performed at the 7th day of hospital admission. Similarly, Drodz (66) indicates that the examination was performed on the 8th day of hospital admission. This inconsistency did not affect the allocation of the studies within a specific phase, and indication reported by the authors was used.

Table S3. General characteristics of the study groups

**Reference**	**Corresponding number in Fig 2 and Fig 5**	**Country**	**Study Design**	**Setting**	**Age (years)[1]**	**N stroke patients who received the nutritional examination**	**%INC**
Yoo 2008 (2)	1	Korea	Observational	Hospital	64.8 ± 10.3	131	12.20
Gomes 2016 (3)	2	UK	Observational	Hyperacute stroke units in hospital	74.7	537	36.30
Davis 2004 (32)	3	Australia	Observational	Hospital	<75 (n=93), ≥75 (n=92)	185	16.20
Kokura 2016 (33)	4	Japan	Observational	Acute care hospitals: geriatric stroke patients	≥ 75	540	11.10
Nozoe 2016 (34)	5	Japan	Observational	Hospital	70.3 ± 12.8	31	6.50
Nip 2011 (8)	6	Australia	Observational	Hospital	69 ± 15	100	73.00

Table S3. General characteristics of the study groups (continued)

Sremanakova 2019 (35)	7	UK	Observational	Stroke center	≥ 18 (n=1101)	727	21.40
Diendéré 2020 (36)	8	Burkina Faso	Observational	Hospitals	60.5 ± 14.0	222	25.20
Vajpayee 2008 (37)	9	India	Observational	Hospital	≥ 28	95	45.30
Gandolfo 2019 (38)	10	Italy	Observational	Stroke unit	72.6 ± 15.5	249	24.50
Crary 2006 (39)	11	US	Observational	Stroke unit in tertiary care hospital	66.2 ± 11.8	76	26.30
NanZhu 2019 (40)	12	China	Observational	Stroke unit	77.94 ± 5.55	324	100.00
Zheng 2015 I: (intervention) (41)	13	China	Experimental	Stroke unit	71.4 ± 9.3	75	17.30

Table S3. General characteristics of the study groups (continued)

Zheng 2015 II: (control) (41)	14	China	Experimental	Ward	71.8 ± 10.1	71	15.50
Shen 2011 (42)	15	Taiwan	Observational	Hospital	70.7 ± 10.3	483	19.70
Food Trial 2005(b) (43)	16	UK	Experimental	Hospital	71±13 (n=2007) and 71±12 (n=2016)	4023	7.80
Xiang 2020 (44)	17	China	Observational	Hospital	66 ± 16	405	36.30
Kokura 2020 (45)	18	Japan	Observational	Acute care hospitals	76.3 ± 12	702	8.30
Barrio 2020 (46)	19	Spain	Observational	Hospital neurology department	≥ 34 (n=166)	164	14.00
Otsuki 2020 (47)	20	Japan	Experimental	Hospital	≥ 65	211	60.70

Table S3. General characteristics of the study groups (continued)

Robertson 2020 (48)	21	Australia	Observational	Acute stroke unit	76±12.8 (n-30) and 76.7±12.1 (n=30)	60	5.00
López Espuela 2019 (49)	22	Spain	Observational	Hospital neurology unit	77.7 ± 7.0	164	35.40
Aliasghari 2019 (50)	23	Iran	Observational	Hospital neurology units	74.42 ± 7.8	253	76.70
Crary 2013 (51)	24	US	Observational	Joint commission-certified primary stroke center in a tertiary-care academic hospital.	65.7	67	32.00
Çoban 2019 I (age group ≥ 65) (52)	25	Turkey	Observational	Hospital	≥ 65 (n=318)	236	66.10
Çoban 2019 II (age group <65) (52)	26	Turkey	Observational	Hospital	< 65 (n=318)	82	12.20
Schwarz 2017 (53)	27	Australia	Observational	Hospital	≥ 18 (n=83)	55	20.00

Table S3. General characteristics of the study groups (continued)

Porter 2019 (54)	28	Australia	Observational	Hospital: acute ward and rehabilitation	≥ 18	994	61.20
Pandian 2011 (55)	29	India	Observational	Hospital	58.1 ± 13.7	448	27.20
Mosselman 2013 (56)	30	The Netherlands	Observational	Stroke unit	65 ± 16.7	73	19.20
Martineau 2005 (57)	31	Australia	Observational	Acute stroke unit	72 ± 12.9	73	19.20
Ha 2011 (58)	32	Norway	Experimental	Hospital acute care ward	≥ 65	344	54.10
Food Trial 2005(a) (59)	33	UK	Experimental	Hospital	Adult population (n=1180)	859	8.60
Medin 2011 (60)	34	Sweden	Observational	Acute stroke unit	Adult population (n=104)	93	71.00

Table S3. General characteristics of the study groups (continued)

Isono 2017 (61)	35	Japan	Observational	Hospital	74.32 ± 0.93	117	17.90
Far 2018 (62)	36	Iran	Observational	Neurology department	65 ± 10 well-nourished group and 63 ± 12.01 malnourished group	380	83.70
Brynningsen 2007 (63)	37	Denmark	Observational	Hospital acute stroke unit and geriatric stroke rehabilitation unit in hospital	77.9±6.9	89	34.80
Kokura 2018 (64)	38	Japan	Observational	Hospital acute care : geriatric stroke inpatients	79.6 ± 7.6	192	10.90
Kang 2020 (65)	39	Korea	Observational	Hospital	67	1906	26.70
Drozdz 2014 (66)	40	Brasil	Observational	Hospital	≥ 35 (n=18)	12	88.30

Table S3. General characteristics of the study groups (continued)

Cai 2020 (67)	41	China	Observational	Hospital	67.8	572	45.80
Naito 2020 (68)	42	Japan	Observational	Hospital	Adult population (n=1915)	1881	13.50
Hirano 2020 (69)	43	Japan	Observational	Rehabilitation hospital	62.7 ± 11.6	80	50.00
Nishioka 2020(b) (70)	44	Japan	Observational	Rehabilitation wards in rehabilitation hospital	78.1 ± 7.9	420	29.80
Nishioka 2020(a) (71)	45	Japan	Observational	Rehabilitation wards of rehabilitation hospital	≥ 66	113	39.80
Kampman 2015 I (72)	46	Norway	Experimental	Stroke unit	≥ 20 (n=199)	121	31.40
Kampman 2015 II (72)	47	Norway	Experimental	Stroke unit	≥ 22 (n=86)	69	24.60

Table S3. General characteristics of the study groups (continued)

Zhang 2015 (73)	48	China	Observational	Hospital	≥ 18	760	3.80
Shiraishi 2018 (74)	49	Japan	Observational	Rehabilitation ward in rehabilitation hospital	72.2 ± 12.5	202	88.10
Hsieh 2017 (75)	50	Taiwan	Observational	Hospital	64.3 ± 11.1	231	66.20
Falsetti 2009 (76)	51	Italy	Observational	Neuro rehabilitation unit	79.4 ± 6.2	151	87.40
Sato 2019 (77)	52	Japan	Observational	Stroke care unit and neurology ward in hospital	77 ± 7	205	42.00
Lim 2010 (78)	53	Korea	Observational	University medical center	61.2 ± 12.5 well-nourished, 66.5 ±1 1.1 moderately malnourished, 64.7 ± 11.2 severely malnourished	73	74.00

Table S3. General characteristics of the study groups (continued)

James 2005 (79)	54	US	Observational	Rehabilitation center	≥ 18 (n=919)	428	78.70
Nishioka 2016 (80)	55	Japan	Observational	Rehabilitation ward	71.6 ± 13.3	897	98.70
Aadal 2015 (81)	56	Denmark	Observational	Rehabilitation hospital	≥ 27	48	33.00
Aquilani 1999 (10)	57	Italy	Observational	Rehabilitation center	60 ± 11	150	30.00
Nishioka 2017 (82)	58	Japan	Observational	Rehabilitation ward	78.5 ± 7.5	264	93.20
Garbagnati 2009 (83)	59	Italy	Experimental	Rehabilitation hospital	65.3 ± 12.9	72	48.40
Westergren 2001 (84)	60	Sweden	Observational	Rehabilitation hospital	78.6	162	12.30

Table S3. General characteristics of the study groups (continued)

Poels 2006 (85)	61	The Netherlands	Observational	Rehabilitation center	56.7 ±11.0	69	34.80
Hama 2005 (86)	62	Japan	Observational	Rehabilitation hospital	66.7 ± 10.2	51	56.90
Maruyama 2018 (87)	63	Japan	Observational	Rehabilitation hospital	63.9 ± 11.0	138	27.00
Shimizu 2019 (88)	64	Japan	Observational	Rehabilitation wards of rehabilitation hospital	78.9 ± 7.7	188	64.90
Carlsson 2012 (89)	65	Sweden	Observational	Stroke unit at hospital, nursing homes and rehabilitation units and a home health service team	83 ± 6.9	15	100.00
Tsai 2008 (90)	66	Taiwan	Observational	Long term care homes / at home	≥ 40	74	81.10

Table S3. General characteristics of the study groups (continued)

Kaur 2008 (91)	67	Australia	Observational	Hospital	73	83	56.60
Jung 2020 (92)	68	Korea	Observational	Department of rehabilitation medicine at tertiary hospital	66.8 ± 15.3	40	37.50
Van Zwienen-Pot 2017 (93)	69	The Netherlands	Observational	Nursing home rehabilitation wards	≥ 65 y	26	46.00
Campillo 2004 (94)	70	France	Observational	Rehabilitation care hospital: neuro-disease department	71.0 ± 11.7	85	48.20
Da Silva 2019 (95)	71	Brazil	Observational	Hospital	Adult population	34	55.90
Lelli 2019 (96)	72	Italy and Spain	Observational	Hospital geriatric rehabilitation unit	≥ 65	127	58.00
Scrutinio 2020 (97)	73	Italy	Observational	Inpatient rehabilitation facility	≥ 67	668	24.30

Table S3. General characteristics of the study groups (continued)

Perry 2004 (11)	74	UK	Observational	Home	71 ± 12.9	206	12.10
Vilardell 2017 (98)	75	Spain	Experimental	Hospital	Adult population (n=225)	219	27.80
Westergren 2008 (99)	76	Sweden	Observational	Home, special accommodation, other settings	77.2 ± 6.6	89	41.00
Choi 2015 (100)	77	Korea	Observational	Medical center	Adult population (n=203)	196	83.70
Kim 2013 (101)	78	Korea	Observational	Hospital rehabilitation department	70.6 ± 7.2	35	91.40

[1] age refers to the number of patients who received the nutritional examination unless otherwise indicated

Figure S2. Pooled prevalence of INC per continent

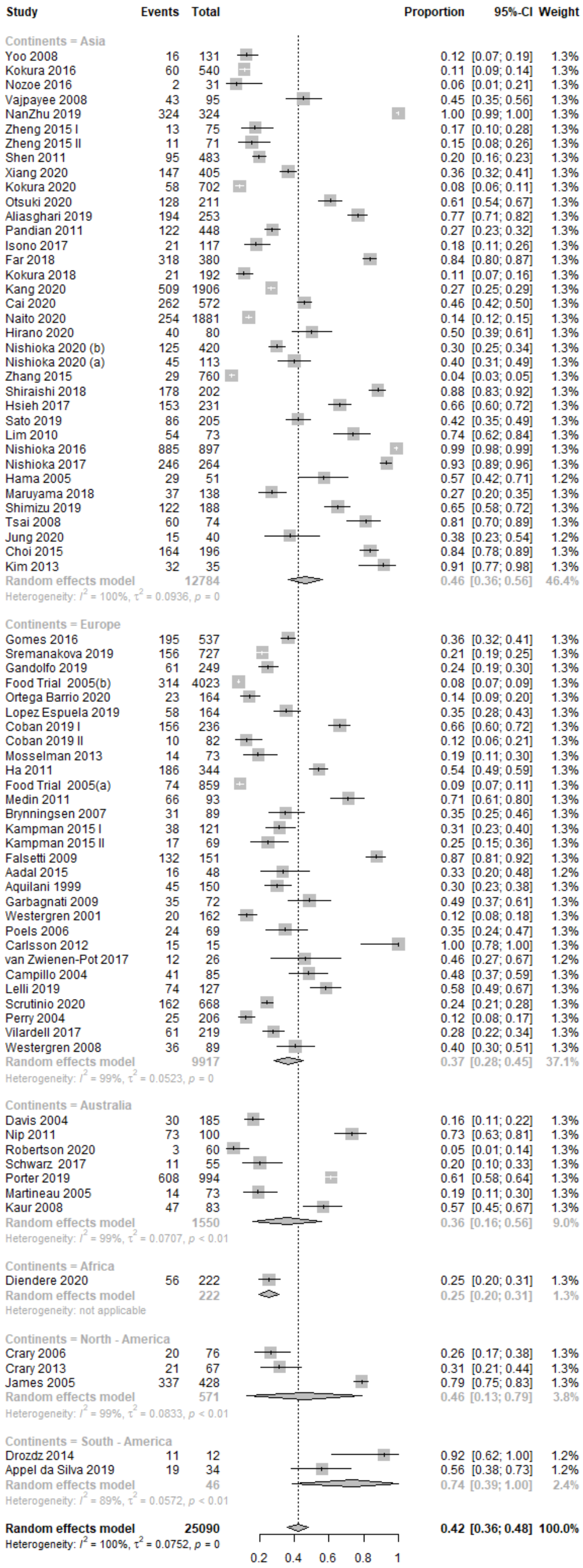

Table S4. Risk of bias per study group

Reference	Corresponding number in Fig 2 and Fig 5	Selection	Performance I	Perfomance II	Detection I	Detection II	Reporting I	Reporting II	Relative risk (%)
Yoo 2008 (2)	1								
Gomes 2016 (3)	2								
Davis 2004 (32)	3								
Kokura 2016 (33)	4								
Nozoe 2016 (34)	5								
Nip 2011 (8)	6								
Sremanakova 2019 (35)	7								
Diendéré 2020 (36)	8								
Vajpayee 2008 (37)	9								
Gandolfo 2019 (38)	10								
Crary 2006 (39)	11								
NanZhu 2019 (40)	12								
Zheng 2015 I: (intervention) (41)	13								
Zheng 2015 II: (control) (41)	14								
Shen 2011 (42)	15								
Food Trial 2005(b) (43)	16								
Xiang 2020 (44)	17								
Kokura 2020 (45)	18								

Barrio 2020 (46)	19
Otsuki 2020 (47)	20
Robertson 2020 (48)	21
López Espuela 2019 (49)	22
Aliasghari 2019 (50)	23
Crary 2013 (51)	24
Çoban 2019 I (age group ≥ 65) (52)	25
Çoban 2019 II (age group <65) (52)	26
Schwarz 2017 (53)	27
Porter 2019 (54)	28
Pandian 2011 (55)	29
Mosselman 2013 (56)	30
Martineau 2005 (57)	31
Ha 2011 (58)	32
Food Trial 2005(a) (59)	33
Medin 2011 (60)	34
Isono 2017 (61)	35
Far 2018 (62)	36
Brynningsen 2007 (63)	37
Kokura 2018 (64)	38
Kang 2020 (65)	39
Drozdz 2014 (66)	40
Cai 2020 (67)	41
Naito 2020 (68)	42
Hirano 2020 (69)	43

Nishioka 2020(b) (70)	44								
Nishioka 2020(a) (71)	45								
Kampman 2015 I (72)	46								
Kampman 2015 II (72)	47								
Zhang 2015 (73)	48								
Shiraishi 2018 (74)	49								
Hsieh 2017 (75)	50								
Falsetti 2009 (76)	51								
Sato 2019 (77)	52								
Lim 2010 (78)	53								
James 2005 (79)	54								
Nishioka 2016 (80)	55								
Aadal 2015 (81)	56								
Aquilani 1999 (10)	57								
Nishioka 2017 (82)	58								
Garbagnati 2009 (83)	59								
Westergren 2001 (84)	60								
Poels 2006 (85)	61								
Hama 2005 (86)	62								
Maruyama 2018 (87)	63								
Shimizu 2019 (88)	64								
Carlsson 2012 (89)	65								

Tsai 2008 (90)	66								
Kaur 2008 (91)	67								
Jung 2020 (92)	68								
Van Zwienen-Pot 2017 (93)	69								
Campillo 2004 (94)	70								
Da Silva 2019 (95)	71								
Lelli 2019 (96)	72								
Scrutinio 2020 (97)	73								
Perry 2004 (11)	74								
Vilardell 2017 (98)	75								
Westergren 2008 (99)	76								
Choi 2015 (100)	77								
Kim 2013 (101)	78								

Table S4. Legend

Risk of bias

No risk of bias

Risk of bias is not applicable
